# Finite Element Analysis of Osteoporotic and Osteoblastic Vertebrae and Its Association With the Proton Density Fat Fraction From Chemical Shift Encoding-Based Water-Fat MRI – A Preliminary Study

**DOI:** 10.3389/fendo.2022.900356

**Published:** 2022-07-11

**Authors:** Tobias Greve, Nithin Manohar Rayudu, Michael Dieckmeyer, Christof Boehm, Stefan Ruschke, Egon Burian, Christopher Kloth, Jan S. Kirschke, Dimitrios C. Karampinos, Thomas Baum, Karupppasamy Subburaj, Nico Sollmann

**Affiliations:** ^1^ Department of Neurosurgery, University Hospital, Ludwig-Maximilians-University (LMU) Munich, Munich, Germany; ^2^ Department of Diagnostic and Interventional Neuroradiology, School of Medicine, Klinikum rechts der Isar, Technical University of Munich, Munich, Germany; ^3^ Engineering Product Development (EPD) Pillar, Singapore University of Technology and Design (SUTD), Singapore, Singapore; ^4^ Department of Diagnostic and Interventional Radiology, School of Medicine, Klinikum rechts der Isar, Technical University of Munich, Munich, Germany; ^5^ Department of Diagnostic and Interventional Radiology, University Hospital Ulm, Ulm, Germany; ^6^ TUM-Neuroimaging Center, Klinikum rechts der Isar, Technical University of Munich, Munich, Germany; ^7^ Sobey School of Business, Saint Mary’s University, Halifax, NS, Canada

**Keywords:** finite element analysis, osteoporosis, metastasis, vertebral fractures, bone mineral density, magnetic resonance imaging, proton density fat fraction, spinal neoplasms

## Abstract

**Purpose:**

Osteoporosis is prevalent and entails alterations of vertebral bone and marrow. Yet, the spine is also a common site of metastatic spread. Parameters that can be non-invasively measured and could capture these alterations are the volumetric bone mineral density (vBMD), proton density fat fraction (PDFF) as an estimate of relative fat content, and failure displacement and load from finite element analysis (FEA) for assessment of bone strength. This study’s purpose was to investigate if osteoporotic and osteoblastic metastatic changes in lumbar vertebrae can be differentiated based on the abovementioned parameters (vBMD, PDFF, and measures from FEA), and how these parameters correlate with each other.

**Materials and Methods:**

Seven patients (3 females, median age: 77.5 years) who received 3-Tesla magnetic resonance imaging (MRI) and multi-detector computed tomography (CT) of the lumbar spine and were diagnosed with either osteoporosis (4 patients) or diffuse osteoblastic metastases (3 patients) were included. Chemical shift encoding-based water-fat MRI (CSE-MRI) was used to extract the PDFF, while vBMD was extracted after automated vertebral body segmentation using CT. Segmentation masks were used for FEA-based failure displacement and failure load calculations. Failure displacement, failure load, and PDFF were compared between patients with osteoporotic vertebrae versus patients with osteoblastic metastases, considering non-fractured vertebrae (L1-L4). Associations between those parameters were assessed using Spearman correlation.

**Results:**

Median vBMD was 59.3 mg/cm^3^ in osteoporotic patients. Median PDFF was lower in the metastatic compared to the osteoporotic patients (11.9% *vs*. 43.8%, p=0.032). Median failure displacement and failure load were significantly higher in metastatic compared to osteoporotic patients (0.874 mm *vs*. 0.348 mm, 29,589 N *vs*. 3,095 N, p=0.034 each). A strong correlation was noted between PDFF and failure displacement (rho -0.679, p=0.094). A very strong correlation was noted between PDFF and failure load (rho -0.893, p=0.007).

**Conclusion:**

PDFF as well as failure displacement and load allowed to distinguish osteoporotic from diffuse osteoblastic vertebrae. Our findings further show strong associations between PDFF and failure displacement and load, thus may indicate complimentary pathophysiological associations derived from two non-invasive techniques (CSE-MRI and CT) that inherently measure different properties of vertebral bone and marrow.

## Introduction

Osteoporosis is a highly prevalent disease that imposes enormous costs on individuals and society (1, 2). The estimated prevalence of osteoporosis worldwide is 23.1% in women and 11.7% in men (3). Osteoporosis-related fragility fractures account for high morbidity and represent a high burden on disability-adjusted years of life (4). The spine is among the most frequent sites for those fragility fractures, and affected patients show a more than 10-fold increased risk for future additional vertebral fractures (VFs) (5–7). Furthermore, the spine is also the most common site of bone metastases, accounting for approximately 50% of cases (8). Spinal metastases occur in approximately 5% to 10% of patients with primary cancer, resulting in about 400,000 new cases of bone metastases each year in the United States alone, underscoring high socioeconomic relevance (9–11). The most common cause of osteoblastic metastases is prostate cancer, but other tumor types such as lung cancer, breast cancer, or bladder cancer can cause these lesions as well (12). Like osteoporosis, vertebral metastases lead to an increased risk of VFs, which can cause severe pain, limb dysfunction, and spinal cord compression, thus markedly affecting the patients’ quality of life (1, 13).

The assessment of osteoporosis-related VF risk in routine clinical practice is primarily based on the evaluation of T-scores, which are derived from measurements of areal bone mineral density (aBMD) using dual-energy X-ray absorptiometry (DXA) (14–16). However, DXA is of limited value in identifying patients at high risk of fracture (17–20). Volumetric BMD (vBMD) derived from computed tomography (CT) imaging is only a surrogate measure of bone strength and cannot fully explain fracture incidences (15, 16, 21). In that regard, CT-based finite element analysis (FEA) is a computational approach of generating three-dimensional (3D) patient-specific models that can realistically calculate *in-vivo* material behavior using numerical simulation (22). During biomechanical testing of bones, an increasing strain rate is applied to the bone until failure due to produced deformations or displacements throughout the structure. The load-deformation behavior of the bone is a linear (elastic) region before yield, a post-yield non-linear region containing the maximum (ultimate) load, and the failure point at which bone fracture occurs. During FEA, a simulation is conducted in which a compression loading condition is generated by applying displacement loading on the superior surface. After solving a plotted load versus displacement curve, failure load and failure displacement can be calculated (23). Yeung et al. have observed that using the FEA-predicted failure load and displacement values of the baseline data, it is possible to predict fracture risk in the follow-up fractured vertebrae using CT (24). Specifically, the vertebrae that are going to fail in the future may be characterized by deteriorated bone strength in the baseline data (24). The FEA-derived parameters may have accurately captured the mechanical behavior variation due to the occurrence of follow-up osteoporotic fractures in the vertebrae (24). In osteoporosis, FEA with calculation of the failure load and failure displacement may provide detailed parameters on vertebral bone strength and may more accurately estimate fracture risk than BMD alone (19, 20, 25–28). While being well established in osteoporosis, only a few studies have presented experimentally validated FEA models for bone strength assessment of vertebrae with metastatic lesions. However, such lesions can also severely impact a vertebral body’s structure and resistance to fracture (23, 29).

Besides CT, magnetic resonance imaging (MRI) techniques emerge for assessment of the osteoporotic spine (30, 31). Specifically, the proton density fat fraction (PDFF) of bone marrow as derived from chemical shift encoding-based water-fat MRI (CSE-MRI) provides a map of hydrogen proton density attributable to fat normalized to the total hydrogen proton density and provides an accurate estimate of fat volume fraction (32, 33). The PDFF was shown to facilitate discrimination between benign and malignant lesions because most malignant neoplasms tend to replace the cellular and fatty bone marrow elements, thus resulting in low PDFF values (34–37). In contrast, benign skeletal lesions usually resemble fat in the bone marrow and exhibit higher PDFF values (34–37). In addition, the PDFF has also been implicated as a biomarker for osteoporosis and VF risk (38, 39). In this context, it was shown that PDFF is increased in osteoporosis and negatively correlates with BMD (38, 40–42).

While there is evidence for the applicability of FEA-based parameters derived from CT imaging and PDFF derived from CSE-MRI in osteoporotic and osteoblastic vertebral bodies, the two techniques inherently measure different properties of the vertebral bone and marrow, raising the question of the association between these measures. Therefore, the aim of this study was to compare how accurately osteoporotic and diffuse osteoblastic metastatic changes in lumbar vertebrae can be differentiated based on the abovementioned parameters (vBMD, PDFF, and measures derived from FEA) and how closely these parameters correlate within the same group of patients.

## Materials and Methods

### Study Inclusion and Patient Cohort

This retrospective study was approved by the local institutional review board (ethics committee reference number 5679/13) and was conducted in accordance with the Declaration of Helsinki. Written informed consent was waived due to the study’s retrospective design.

Patients who received a CT and MRI examination of the spine from the standard clinical routine protocol between December 2018 and May 2019 were screened for inclusion. They were identified in our hospital’s picture archiving and communication system (PACS). Inclusion criteria were (1) acquisition of CSE-MRI and CT of the lumbar spine within 30 days, and (2) osteoporosis with or without osteoporotic fractures in the lumbar vertebral bodies L1 to L4, or, alternatively, diffuse osteoblastic lesions in the vertebral bodies L1 to L4. Exclusion criteria were (1) age below 18 years, (2) motion artifacts in imaging data, (3) previous surgery with instrumentation at the lumbar spine, (4) severe degenerative changes including Schmorl nodes or Modic-type endplate changes (grade 3), (5) inflammatory processes with related bone marrow affection (e.g., spondylodiscitis), and (6) pregnant or breastfeeding women. The presence of VFs in L1 to L4 was not an exclusion criterion, but due to changes in PDFF and/or BMD in vertebral bodies upon acute or old VFs, these vertebral bodies were excluded from averaging and further statistical analysis. Overall, seven patients were eligible and included in this study.

### Computed Tomography

#### Image Acquisition

Image acquisition was performed in supine position using multi-detector CT scanners (Brilliance 64, Ingenuity CT, Philips Healthcare, Best, The Netherlands; Somatom Definition AS+, Somatom Sensation Cardiac 64, Siemens Healthineers, Erlangen, Germany). An initial scout scan was used for planning of the field of view, and subsequent helical scanning was acquired with a peak tube voltage of 120 kVp or 130 kVp and adaptive tube load, without previous application of any intravenous or oral contrast agents. Sagittal reformations of the spine with a slice thickness ≤3 mm were reconstructed with a bone kernel and used for further analysis in this study. The sagittal reformations of the spine were used for VF detection by a board-certified radiologist with 11 years of experience, who used the classification proposed by Genant et al. (43). CT imaging was performed for various indications not related to bone densitometry.

#### Extraction of Volumetric BMD

**Figure 1:**
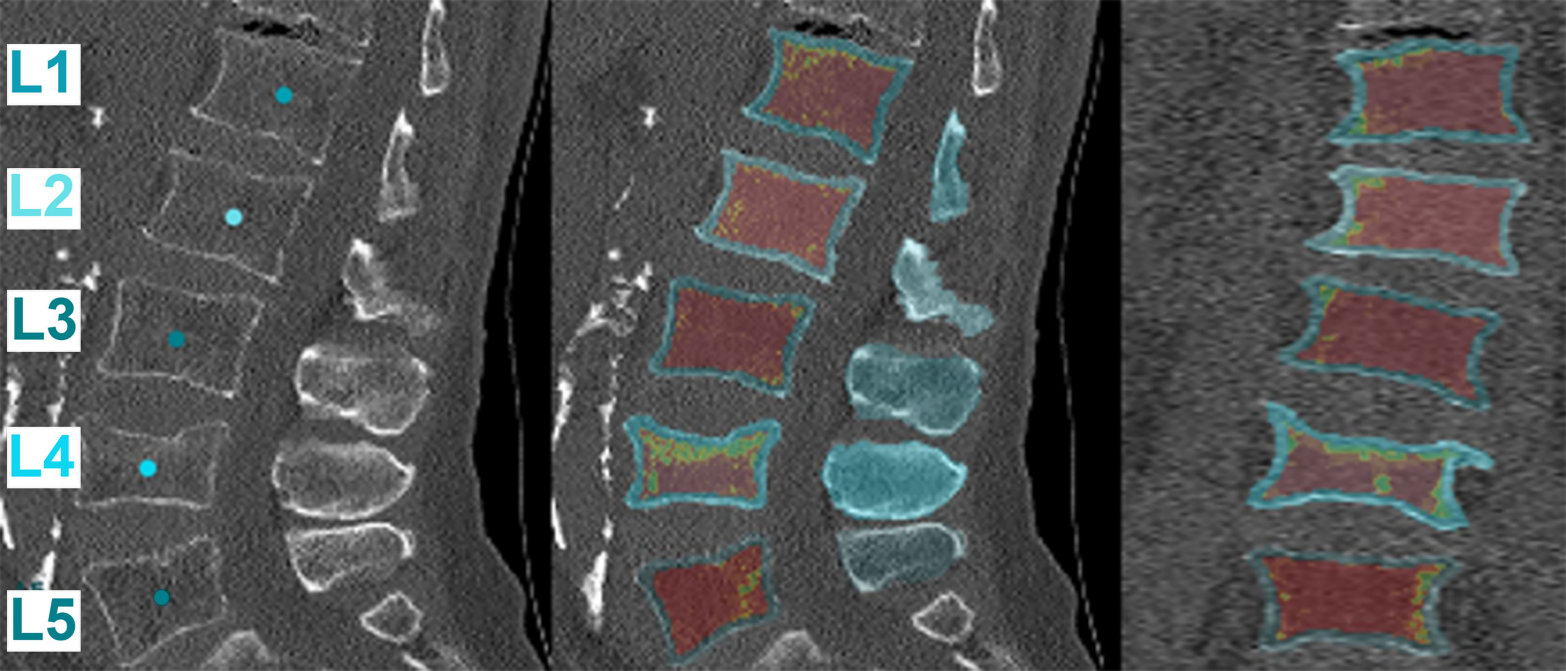
Automatic segmentation and extraction of volumetric bone mineral density (vBMD). The first tile shows the automatic labeling of the vertebral segments. Subsequently, the vBMD is calculated for each vertebral body using different planes (sagittal: middle tile, coronal: right tile). Red marks areas of low vBMD, while green marks areas of high vBMD. Note that fractured vertebrae, in this case L4, have a falsely high vBMD and were excluded from the analysis (https://anduin.bonescreen.de).

Measurements of vBMD were extracted from clinical routine CT scans of the lumbar spine and the median over multiple levels was calculated (L1 to L4, except for fractured vertebrae). Volumetric measures were extracted opportunistically in a semi-automatic multi-step procedure (https://anduin.bonescreen.de) (44–46). First, vertebrae were automatically segmented in CT scans to enclose the entire trabecular compartment using a framework of convolutional neural networks that identifies the spine, labels each vertebral body, and creates segmentation masks, adjusting for the used scanning protocol (120 kVp or 130 kVp) and scanner (44–46). Second, vertebral bodies were separated from posterior elements in these masks using affine and deformable transformations to fit templates of vertebral subregions to each vertebral level (**Figure 1**) (44–46). The vBMD was not extracted from vertebral bodies with fractures or osteoblastic metastases, given that this parameter was previously shown to be falsely elevated in osteoblastic metastases (47). In addition to extraction of vBMD from segmented vertebrae of the lumbar spine, clinical routine CT scans with segmentation masks including the posterior elements were further used for FEA.

#### Finite Element Analysis

The CT data and segmentation masks were imported to the open-source medical imaging software 3D Slicer (https://www.slicer.org; Surgical Planning Laboratory, Brigham and Women’s Hospital, Boston, MA, USA) to reconstruct and generate 3D vertebral models (48). These generated 3D vertebral models were then imported into Abaqus CAE (version 6.10; Dassault Systèmes Simulia Corp., Johnston, RI, USA) for downstream FEA (49). The vertebral models were meshed with linear tetrahedron (C3D4) elements. We used the tetrahedral element for meshing to capture the geometry accurately. The meshed model and CT data were then imported to material mapping software Bonemat (version 3.2; http://www.bonemat.org, Bioengineering and Computing Laboratory, Istituto Ortopedico Rizzoli, Bologna, Italy), which maps on an FEA mesh the bone elastic properties derived from CT images (50). Image attenuation-based material properties were mapped to the meshed vertebral body using Hounsfield unit (HU)-density-modulus relations (**Table 1**). Then, the material mapped model was imported back to Abaqus CAE software for further processing.

**Table 1:** Quantitative paramters for the lumbar spine.

ID	Group	PDFF (%)					Failure Displacement (mm)					Failure Load (N)				
		L1	L2	L3	L4	L1-L4	L1	L2	L3	L4	L1-L4	L1	L2	L3	L4	L1-L4
1	Osteoblastic metastasis	24.1	26.2	27.8	31.8	27.0 [25.7-28.8]	1.027	0.775	0.911	0.724	0.843 [0.762-0.940]	19735	13240	39470	27447	23591 [18111-30453]
2	Osteoblastic metastasis	14.4	10.3	9.4	5.6	9.9 [8.5-11.3]	1.050	0.708	0.804	0.979	0.892 [0.780-0.997]	29731	29447	22665	50760	29589 [27752-34988]
3	Osteoblastic metastasis	7.7	13.4	8.8	9.8	9.3 [8.5-10.7]	0.884	0.841	0.942	0.864	0.874 [0.858-0.899]	45616	53618	53510	27663	49563 [41128-53537]
4	Osteoporosis	46.0	41.4	35.8	49.4	47.7 [46.9-48.6]	0.325	0.978	0.769	0.526	0.426 [0.375-0.476]	4161	3173	10248	3095	3628 [3361-3895]
5	Osteoporosis	33.4	38.0	41.6	26.3	38.0 [35.7-39.8]	0.296	0.369	0.348	0.315	0.348 [0.322-0.359]	3744	4108	4303	5995	4108 [3926-4205]
6	Osteoporosis	41.0	8.1	43.8	38.5	42.4 [41.7-43.1]	0.221	0.224	0.311	0.257	0.266 [0.244-0.289]	3235	6275	2728	6024	2981 [2854-3108]
7	Osteoporosis	43.9	43.0	48.6	45.5	44.7 [43.7-46.2]	0.301	0.502	0.605	0.504	0.503 [0.451-0.529]	2188	2797	2611	2558	2584 [2465-2658]

Grey cells refer to fractured vertebrae. Median and inter-quartile ranges (IQRs) for vertebral bodies L1 to L4 were calculated (without the fractured vertebrae).

**Figure 2:**
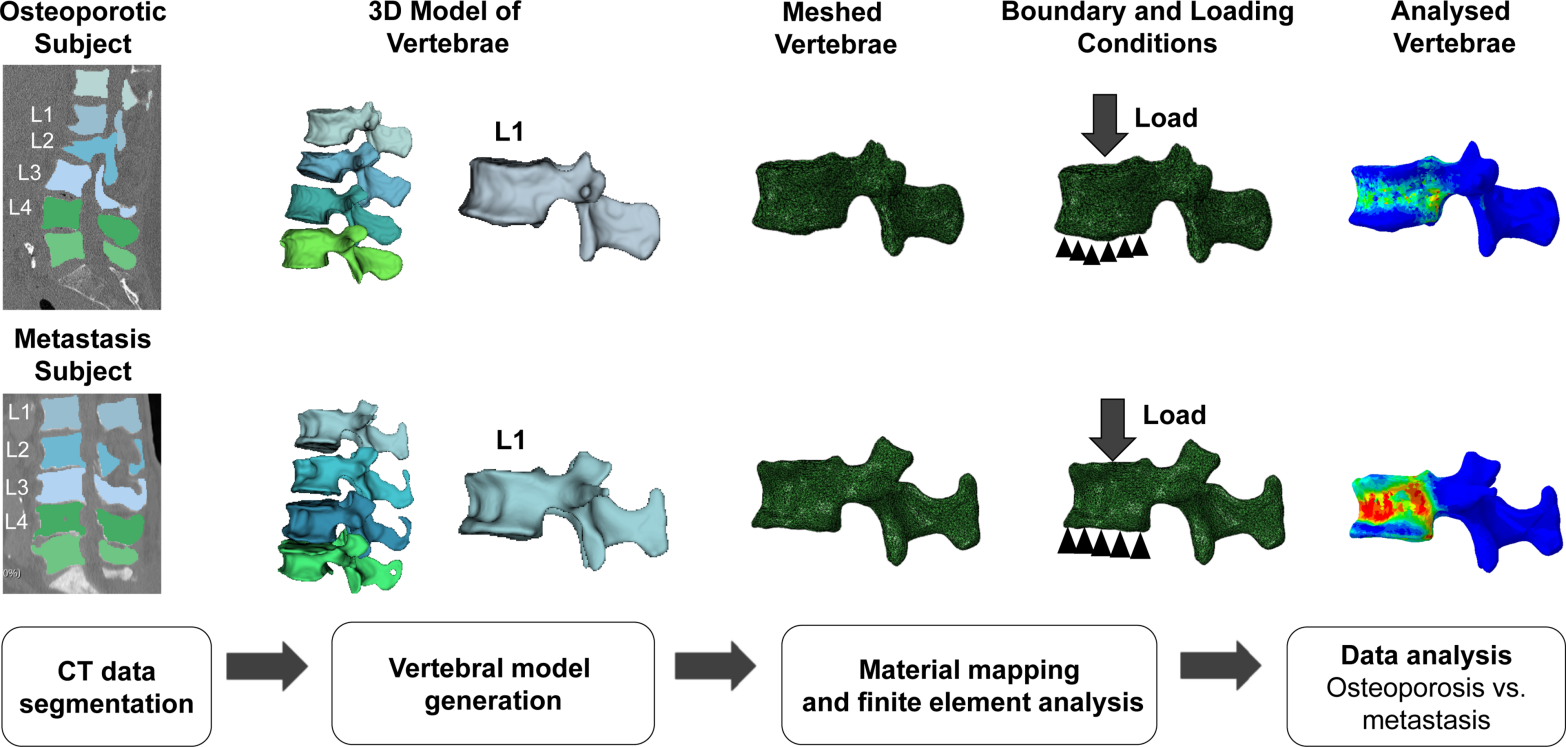
Four-step finite element analysis (FEA) methodology for the calculation of FEA-based failure load and failure displacement in osteoporosis and metastasis models. Schematic representation of the workflow followed for FEA of the models reconstructed from computed tomography (CT) images. The L1 vertebrae model is shown here as a representative example of the analysis of the L1-L4 section. The top and bottom rows of the figure show the analyses for osteoporotic and metastatic vertebrae, respectively. The analysis resulted in the calculation of the FEA-based failure load and displacement for osteoporotic and osteoblastic metastasized vertebrae.

In this study, we simulated the compression loading condition by fixing the inferior surface of the vertebrae and applying the normal displacement loading on the superior surface. After solving, the plotted load versus displacement curve was used to calculate failure load and failure displacement. The FEA methodology used in the current study has been validated experimentally in previous studies (**Figure 2**; **Table 2**) (27, 57, 58). To maintain the accuracy of the computational model, a mesh convergence assessment was carried out by varying the element edge length from 1 to 3 mm, with an increment of 0.5 mm. This assessment showed that 2 mm element size gives the mesh independent results, and the same size was chosen to mesh all the vertebral models for downstream analysis.

**Table 2:** Material mapping relations used in the current study for the calculation of failure load and displacement.

Property	Mapping Relations
*Apparent density (ρ_app_ in Kg/m^3^)* (51)	ρ_app_ = 47 + 1.122 × HU HU-Hounsfield unit
*Ash density (ρ_ash_ in Kg/m^3^)* (52)	ρ_ash_= 0.6 × ρ_app_
*Elastic modulus (E in MPa)* (51, 53)	E_z_ = 4730 × (ρ_app_)^1.56^ E_x_= E_y_ = 0.333 E_z_ Z-axial direction of the vertebra
*Shear modulus (G in MPa)* (54)	G_xy_ = 0.121 E_z_ G_xz_ = G_yz_ = 0.157 E_z_
*Poisson ratio (V)* (54)	Vxy = 0.381 Vxz = Vyz = 0.104
*Maximum principal stress limit (σ in MPa)* (55)	σ = 137 × ρ_ash_^1.88^, ρ_ash_ < 0.317 σ = 114 × ρ_ash_^1.72^, ρ_ash_ > 0.317
*Plastic strain (ϵ_AB_)* (56)	ϵ_AB_ = -0.00315 + 0.0728 ρ_ash_
*Minimum principal stress limit (σ_min_ in MPa)* (56)	σ_min_ = 65.1 × ρ_ash_^1.93^

### Magnetic Resonance Imaging

#### Image Acquisition

Acquisition of MRI of the lumbar spine was performed in the supine position on a 3-Tesla scanner (Release 5.4; Ingenia, Philips Healthcare, Best, The Netherlands) using a monopolar time-interleaved multi-echo gradient echo sequence, acquiring 6 echoes in 2 interleaves with 3 echoes per interleave (59). For all patients, the imaging parameters were set to TEmin = 1.12 ms, deltaTE= 0.96 ms, orientation = sagittal, readout direction = anterior-posterior, approximate field of view = 219.6 x 219.6 x 79.2 mm^3^, with an isotropic acquisition voxel size of 1.8 mm. A sagittal T1-weighted turbo spin echo sequence (TR/TE = 600/8 ms) and a sagittal T2-weighted turbo spin echo DIXON sequence (TR/TE: 2,500/100 ms) of the lumbar spine, together with axial acquisitions over selected areas, were added for clinical purposes.

#### Image Processing

Fat quantification was performed offline using a complex-based water-fat separation, estimating the field map using a variable-layer single-min-cut graph-cut technique (60). The water-fat signal model was solved including a precalibrated seven-peak fat spectrum and a single T2* to model the signal variation with TE (61, 62). The PDFF maps were computed as the ratio of the fat signal over the sum of fat and water signals (32, 33). The vertebral bodies L1 to L4 were included in the analysis and manually segmented by a radiologist with 3 years of experience in spine imaging. Segmentation was performed on the PDFF maps using the open-source software MITK (http://mitk.org/wiki/The_Medical_Imaging_Interaction_Toolkit_(MITK); German Cancer Research Center, Division of Medical and Biological Informatics, Medical Imaging Interaction Toolkit, Heidelberg, Germany) (63). PDFF values were calculated individually for each segmented vertebra from L1 to L4. The median of these PDFF values was calculated without the fractured vertebrae.

### Statistics

The statistical analyses were performed with SPSS software (version 26; IBM SPSS Statistics for Windows, IBM Corp., Armonk, NY, USA). Tests were performed using a two-sided level of significance of α = 0.05.

Except for descriptive statistics, fractured vertebrae were excluded from further analysis and averaging. According to Shapiro-Wilk tests, the data distribution of the majority of measures of this study was non-parametric. Thus, Friedman’s two-way analysis of variance by ranks was performed to test whether there were statistically significant differences among the ≤4 vertebral bodies per patient for the investigated variables. Subsequently, the median and inter-quartile ranges (IQRs) for PDFF, failure displacement, and failure load were calculated from L1 to L4. Mann-Whitney U test was performed to test for differences between patients with osteoblastic metastasis (metastasis group) and low bone density (osteoporosis group) regarding PDFF, failure displacement, and failure load. Spearman’s rho with reporting of 95% confidence intervals (CIs) was used to correlate PDFF with failure displacement and failure load. Furthermore, Spearman’s rho (with 95% CIs) was also used to investigate associations of vBMD, PDFF, failure displacement, and failure load in osteoporotic patients.

## Results

### Clinical and Radiographic Characteristics

**Figure 3:**
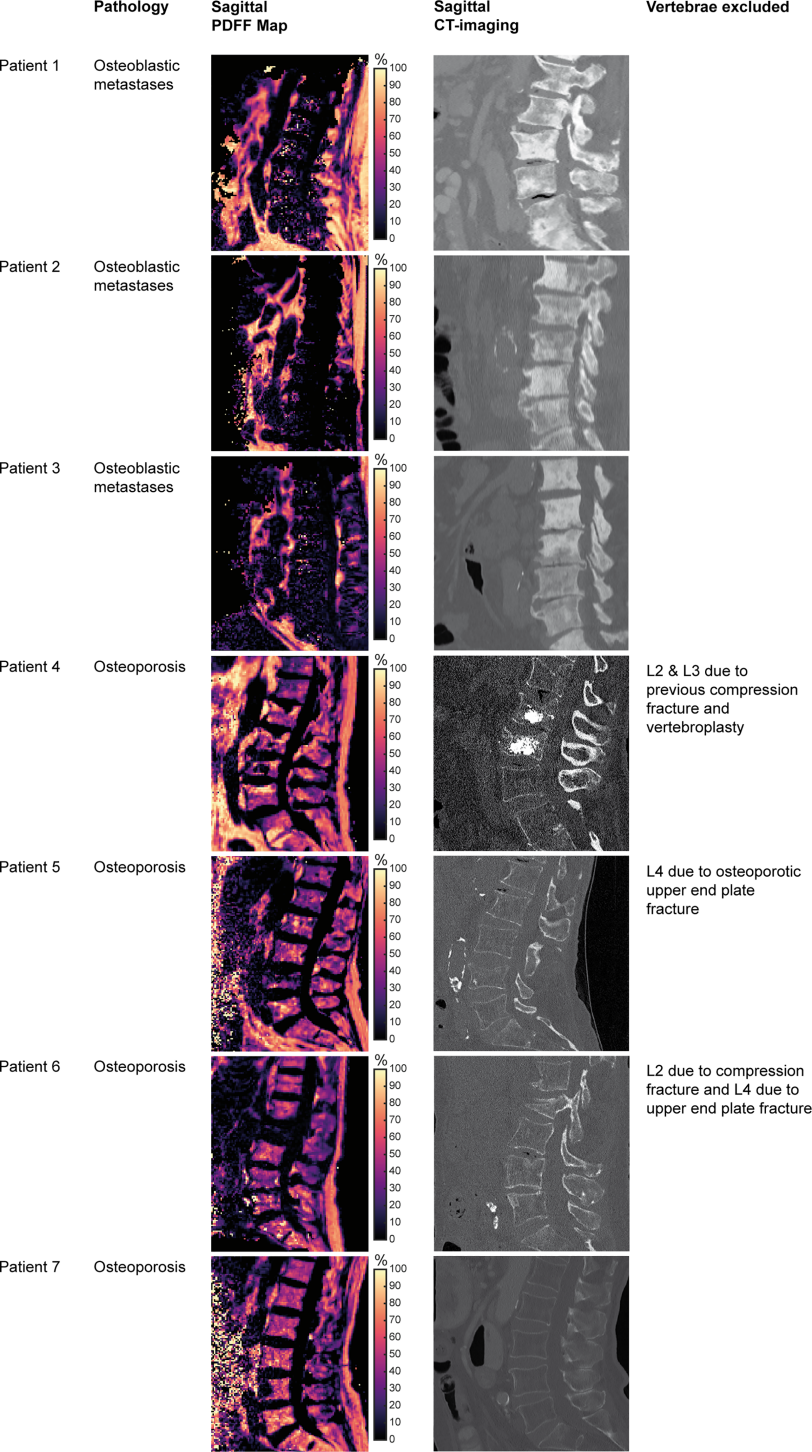
Patient characteristics and imaging. Tiles showing vertebral bodies L1 to S1 on sagittal reformations of proton density fat fraction (PDFF) maps obtained from the six-echo monopolar time-interleaved multi-echo gradient-echo sequence and CT images, respectively.

We included seven patients in the analysis (4 males and 3 females), with a median age of 77.5 years (**Figure 3**). One group consisted of three patients that showed diffuse osteoblastic metastatic changes in the lumbar vertebrae L1 to L4 (metastasis group). None of these patients suffered from fractured vertebrae in these levels. In detail, this group was composed of one 81-year-old female with unilateral breast cancer (initial diagnosis 2006, bone metastases known since 2015, initial treatment with breast-conserving surgery with sentinel lymph node dissection, radiotherapy, and hormonal therapy) and two males (71 and 74 years old) with prostate cancer (initial diagnosis 2015 and 2017, bone metastases known since 2017 in both patients, initial treatment with prostatectomy, chemotherapy, and hormonal therapy in both patients).

The other group consisted of four patients with osteoporosis, with a median vBMD of 59.3 mg/cm^3^ (IQR: 56.4 to 64.9 mg/cm^3^) according to opportunistic measurements in CT (osteoporosis group). The first of these patients (ID 4) had previous osteoporotic VFs and vertebroplasty of L2 and L3. The second of these patients (ID 5) had an osteoporotic upper endplate fracture of L4. The third patient of this group (ID 6) showed a compression fracture of L2 and an old upper endplate fracture of L4. The last patient of this group (ID 7) did not show any VFs.

### Comparison of Metastatic and Osteoporotic Vertebrae

**Figure 4:**
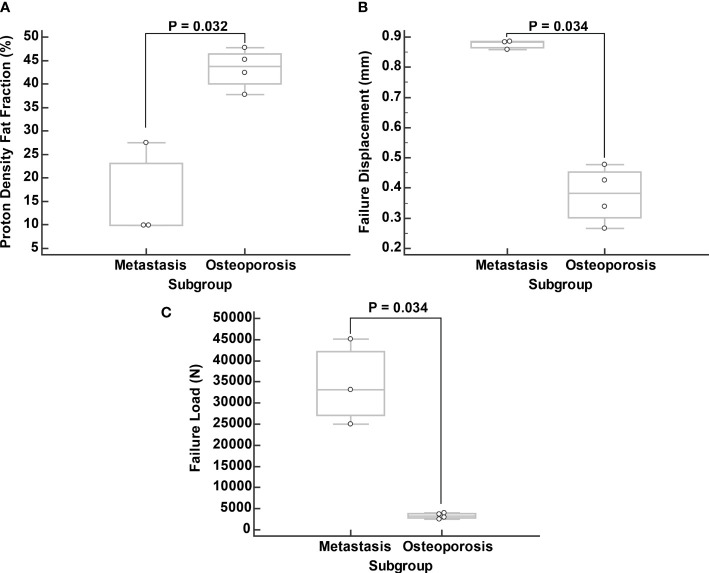
Comparison of proton density fat fraction (PDFF; **A**), failure displacement **(B)**, and failure load **(C)** between subgroups. Boxplots were calculated from median values of vertebral bodies L1 to L4 excluding the fractured vertebrae.

In the metastasis group, the median vertebral PDFF was 11.9% (IQR: 9.3 to 24.7%), while in the osteoporosis group the median PDFF was 43.8% (IQR: 41.3 to 45.7%) (p = 0.032) (**Figure 4**). Outliers in PDFF within the same patient were most evident in the fractured vertebrae, which were already excluded from the analysis a priori. In particular, this was true for vertebral body L2 of patient ID 6, which had a compression fracture for which the PDFF was 8.1%, while it ranged from 38.5% to 43.8% for the other vertebrae (**Table 1**).

Regarding failure displacement, the median value was 0.874 mm (IQR: 0.797 to 0.951 mm) in the metastasis group and 0.348 mm (IQR: 0.306 to 0.503 mm) in the osteoporosis group (p = 0.034) (**Figure 4**). The median failure load was 29,589 N (IQR: 26,252 to 46,902 N) in the metastasis group and 3,095 N (IQR: 2,669 to 3,926 N) in the osteoporosis group (p = 0.034) (**Figure 4**).

### Correlation of PDFF and FEA-Based Parameters

**Figure 5:**
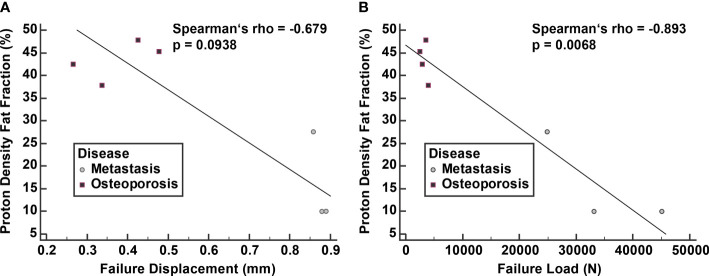
Correlation of proton density fat fraction (PDFF) with failure displacement **(A)** and failure load **(B)**. Spearman correlation was calculated from median values of vertebral bodies L1 to L4 excluding the fractured vertebrae.

A strong negative correlation was noted between PDFF and failure displacement with a Spearman’s rho of -0.679 (95% CI: -0.947 to 0.152, p = 0.094). Furthermore, a very strong negative and statistically significant correlation was noted between PDFF and failure load with a Spearman’s rho of -0.893 (95% CI: -0.984 to -0.427, p = 0.007) (**Figure 5**).

### Correlation of vBMD, PDFF and FEA-Based Parameters in Osteoporotic Patients

In the osteoporotic patients, the correlation of vBMD with PDFF was weak to moderate with a Spearman’s rho of -0.400 (95% CI: -0.983 to 0.912, p = 0.600). The correlation of vBMD with failure displacement and failure load was equally weak to moderate with a Spearman’s rho of -0.400 (95% CI: -0.983 to 0.912, p = 0.600).

## Discussion

This preliminary study used CT for FEA and CSE-MRI to extract vertebral PDFF at the lumbar spine and investigate differences in the quantitative parameters, including failure displacement, failure load, and PDFF between patients with osteoporosis and patients with osteoblastic vertebral lesions. We demonstrated that failure displacement and failure load derived from CT imaging and FEA and PDFF values derived from CSE-MRI showed statistically significant differences between osteoporotic and osteoblastic vertebral bodies. Furthermore, there was a strong correlation between failure displacement and PDFF and a very strong correlation between failure load and PDFF.

PDFF has evolved as a promising non-invasive quantitative marker to assess tissue fat composition. It was shown to be highly accurate and reproducible among MRI vendors, field strengths, and readers, as shown by collinearity and inter-reader agreement (64). Previously, it was shown that the mean PDFF of malignant vertebral bone marrow lesions was significantly lower compared with benign lesions (3.1% *vs*. 28.2%) (65). A PDFF cutoff of 7.8% demonstrated optimal discriminatory power between benign and malignant lesions (65). In addition, PDFF has been shown to have high accuracy in differentiating acute osteoporotic and neoplastic compression fractures of the spine (66). One other study found an optimal PDFF cutoff to discriminate between benign and malignant lesions of 9% (67). Other previous research applied a preconditioned water-fat total field inversion algorithm that could directly estimate the susceptibility map from complex multi-echo gradient echo data for water-fat regions, which may help to better differentiate between osteoblastic and osteolytic changes in patients with metastatic disease as compared to the local field inversion method and a linear total field inversion method (68). In our study, osteoblastic vertebrae had a median PDFF of 11.9%, which is higher than the PDFF previously described for malignant lesions (67). The likely main reason for the observed measurement differences is probably the different measurement method, as we included the entire trabecular component of the vertebral body in the analysis, whereas previous studies mostly evaluated the respective pathology only at individual slices (65, 67). This may explain the higher PDFF in the current study and does not necessarily represent a contradictory result. In addition, diffuse infiltrations have spatial compartments that may have normal cellular and bony structure, which also could result in higher PDFF values on average. In addition, we did not include osteolytic vertebral lesions, which may also partially explain this difference. Another reason could be the small number of subjects in our study, which gives more weight to outliers, especially considering the inherent natural variability of PDFF in spinal lesions and also in healthy individuals, showing a correlation with age and manifesting a fat gradient from cervical to lumbar spinal levels (65, 69, 70). For example, Schmeel and colleagues reported the PDFF in morphologically normal-appearing vertebral bodies to be 55% (mean age 68 years) (65). In contrast, Baum and colleagues reported normal lumbar vertebral body PDFF in young individuals (mean age 26 years) to be 35% (69). Underlining the high variability of PDFF values in different spinal pathologies, it has been recently shown that PDFF was significantly lower in infectious spondylitis compared to erosive endplate changes (4% versus 35%) (71).

In our study, PDFF was markedly increased in patients with osteoporotic vertebrae compared to the metastasis group. This is in line with the literature, which showed that PDFF is negatively correlated to BMD (40, 41). Hence, it might be a useful MRI-derived marker for osteoporosis and bone health (38, 41, 42). However, the correlation between vBMD and PDFF was weak to moderate in our study, which contrasts with the literature, where a stronger correlation (r = 0.64) between both parameters was shown (41). This is most likely explained by the small number of patients in our osteoporotic subgroup. Nevertheless, the median PDFF value in our osteoporosis subgroup was 43.8%, which was lower than the PDFF values of osteoporotic patients in the literature, where a PDFF value of up to 60% is reported (41). In that regard, a recent study showed that PDFF, while correlating with BMD, was significantly higher in patients with osteoporotic/osteopenic vertebrae with VFs than in osteoporotic/osteopenic patients without VFs, even after adjusting for BMD (39). Although correlating with BMD, the authors suggested that PDFF may be an independent predictor for fracture risk in osteoporosis (39). Furthermore, the mean PDFF in osteoporotic/osteopenic vertebrae without VFs was 39% (39), which closely resembles our PDFF values in non-fractured osteoporotic vertebrae.

Considering the complex microstructure of bone, CT imaging and the vBMD calculated from it provide more information for assessing bone quality than the DXA-derived aBMD (44, 72). Notably, DXA is a two-dimensional assessment, thus neglecting the 3D architecture of a vertebral body, and the aBMD only accounts for approximately 60 to 70% of variation in bone strength (73). Yet, patient-specific image-based FEA is considered the reference standard to estimate vertebral strength, having demonstrated to approximate vertebral body compressive strength even better than vBMD according to an *in-vitro* scenario (54). Specifically, vertebral strength measurements derived from FEA have been shown to improve fracture risk calculation and determine treatment efficacy of single segments and functional spinal units (25, 54, 74). Also, the feasibility of performing FEA from routine clinical imaging data to assess fracture risk has been shown (28). In addition, there is evidence that multi-detector CT acquisition with a dose reduction of up to 75% may still enable discriminating between osteoporotic patients with and without VFs (26). One study also showed that the combination of vBMD measurements and FEA derived from routine CT imaging allowed improved prediction of incidental fractures at a vertebral-specific level (24). While the feasibility and utility of FEA for estimating fracture risk of osteoporotic vertebral bodies have been extensively studied, the value of FEA-based parameters in metastatic spinal lesions has been sparsely investigated (23, 29).

However, metastatic changes in vertebral anatomy and structure are of great clinical importance in treating cancer patients, as they are usually the cause of pathological VFs (75, 76). Scores such as the Spinal Instability Neoplastic Score (SINS) have been developed for patients with bone metastases at the spine (77). It categorizes VF risk based on spinal segment, pain, bone quality, radiographic alignment, vertebral body collapse, and postero-lateral involvement of spinal elements (77). However, the reproducibility of its imaging components is suboptimal (78). Thus, its prognostic value in terms of VF risk seems controversial (79). It was shown that FEA-based models provide interesting insights into simulated osteolytic defects (80, 81). However, spinal metastases often present as osteoblastic lesions in prostate and breast cancer (82). Stadelmann et al. investigated osteolytic and osteoblastic metastases in cadaver models and compared FEA models with *in-vitro* compression models (29). They showed that osteoblastic metastases resulted in significantly worse bone tissue properties compared to controls, whereas osteolytic lesions appeared to have a negligible effect, even though osteolytic lesions displayed a lower percentage of mineralized bone tissue in total (29). They mainly attributed these effects to the woven nature of the newly formed bone in osteoblastic lesions and its lower mineralization around the blastic lesions, whereas the material properties of the bone surrounding osteolytic lesions hardly change (29). Another study experimentally measured bone strength (in kN) in cadaveric vertebrae with osteoblastic, osteolytic, and mixed vertebral metastases (83). The authors showed that vBMD was highly variable in osteoblastic and mixed vertebrae while it was generally reduced in osteolytic vertebrae. They also showed that only vBMD, but not lesion type, was an independent predictor of vertebral bone strength (83). Furthermore, *in-vitro* vertebral strength, measured by compression until failure in a laboratory compression model, was strongly associated with FEA-based strength (r = 0.78) and only moderately associated with bone mineral content (r = 0.66), independent of the lesion type (29). Another preliminary study evaluated FEA models on a vertebra-specific level in three fresh-frozen human donors with multiple myeloma and vertebral compression fractures (23). The authors showed that by applying the same universal loading condition to the vertebral segments T1 to L5, the differences in structural strength highly correlated between *in-vitro* samples and FEA-derived values (23). This study also suggested that absolute fracture load values have little predictive value, while the relative fracture loads provided valuable information on the relative stability between segments (23).

While the PDFF is a quantitative measure of fat content of bone marrow (30, 32, 33), failure load and failure displacement take into account the trabecular morphology including quality factors like bone shape, morphology, critical locations, and bone mass distribution (22, 24). Because these parameters inherently measure different features, it is interesting that they correlate strongly with each other. The most likely reason for this strong correlation is that bone marrow fat content, bone trabecular volume and microarchitecture are in turn also correlated with each other. In this regard, bone marrow fat accumulation is an age-dependent process replacing hematopoietic with fatty bone marrow, but it is also associated with a reduction in BMD (84, 85). Given that adipocytes and osteoblasts share the common precursor mesenchymal stem cells in the bone marrow (86), decreased bone formation observed during aging or osteoporosis may be the result of a disturbance in the equilibrium between adipogenesis versus osteoblastogenesis (84, 87). Increased bone marrow fat deposition was hypothesized to be linked to lower BMD and increased VF risk through a shift in mesenchymal stem cell lineage allocation that favors adipocytes over osteoblasts, leading to reductions in BMD and changes in bone microarchitecture (84, 88).

Empirical evidence of the increased bone marrow fat content in osteoporosis and its association with VF risk was shown for different modalities, such as CSE-MRI (39), MR spectroscopy (42, 89–91), and in biopsy studies (92). Interestingly, higher marrow fat deposition was found to be associated with VF risk, even after adjusting for trabecular BMD (93). Apart from these pathophysiologic considerations, it is important to conduct further studies with larger patient samples to elucidate the question of whether marrow fat content, trabecular and vertebral morphology and BMD are independent factors relevant to VF risk, or whether one of these parameters might be the dominant explanatory factor in osteoporosis.

The limitations of this study are mainly the small sample size of seven patients, which did not allow for an age- and gender-matched study design. In addition, no osteolytic metastases were included in the cohort, which would have added value to the quantitative parameters derived from CT and MRI. For comparison, a cohort of healthy control subjects would have been helpful, also to assess how FEA-based parameters and PDFF vary between vertebrae within a healthy individual for the herein used setup. Furthermore, inter-reader variability in the generation of segmentation maps is a potential pitfall. In our study, this limitation was circumvented for the calculation of vBMD and FEA-based parameters by using deep learning-based segmentation through our standardized pipeline. Another limitation of FEA itself is the still high computational effort. As a result, this technology has not yet been integrated into everyday clinical practice.

## Conclusion

The failure displacement and failure load calculated from CT-based FEA were significantly higher in osteoblastic lumbar vertebral bodies than in osteoporotic lumbar vertebral bodies. Conversely, the PDFF calculated from CSE-MRI was significantly lower in diffuse osteoblastic metastatic vertebral bodies than in osteoporotic vertebral bodies. There was a strong correlation between failure displacement and PDFF and a very strong correlation between failure load and PDFF. We were able to show in a preliminary dataset that PDFF and FEA-based failure load and failure displacement are strongly inversely correlated. As a prospect for future diagnostic application of these modalities, a computationally intensive FEA could be performed in a two-stage opportunistic screening approach for those cases found to have significantly reduced PDFF.

## Data Availability Statement

The raw data supporting the conclusions of this article will be made available by the authors, without undue reservation.

## Ethics Statement

The studies involving human participants were reviewed and approved by the Ethikkommission der Fakultät für Medizin der Technischen Universität München. Written informed consent was waived due to the study’s retrospective design.

## Author Contributions

Conceptualization was performed by TB, KS, and NS. Methodology involved NR, MD, CB, SR, EB, and NS. Software involved NR, MD, DK, TB, KS, and NS. Formal analysis performed by TG, NR, TB, and NS. Investigation was performed by TG and NS. Resources provided by JK, DK, TB, and KS. Data curation was performed by TG and NS. Writing – original draft preparation was performed by TG and NS. Writing – review and editing was performed by all authors. Visualization was performed by TG, NR, MD, KS, and NS. Supervision was performed by TB, KS, and NS. Project administration was performed by TB and NS. All authors contributed to the article and approved the submitted version.

## Funding

The present work was supported by the German Research Foundation (Deutsche Forschungsgemeinschaft, DFG, project 432290010, JK and TB) and the German Society of Musculoskeletal Radiology (Deutsche Gesellschaft für Muskuloskelettale Radiologie, DGMSR, MD and NS).

## Conflict of Interest

The authors declare that the research was conducted in the absence of any commercial or financial relationships that could be construed as a potential conflict of interest.

## Publisher’s Note

All claims expressed in this article are solely those of the authors and do not necessarily represent those of their affiliated organizations, or those of the publisher, the editors and the reviewers. Any product that may be evaluated in this article, or claim that may be made by its manufacturer, is not guaranteed or endorsed by the publisher.

## Abbreviations

3D, three-dimensional; aBMD, areal bone mineral density; CI, confidence interval; vBMD, volumetric bone mineral density; CSE-MRI, chemical shift encoding-based water-fat MRI; CT, computed tomography; DXA, dual-energy X-ray absorptiometry; FEA, finite element analysis; MRI, magnetic resonance imaging; IQR, inter-quartile range; PDFF, proton density fat fraction; VF, vertebral fracture; PACS, picture archiving and communication system; HU, Hounsfield unit; SINS, Spinal Instability Neoplastic Score.
